# Morphologic analysis of macular neovascularizations by OCT angiography—Technical limitations in the comparison of 3×3mm and 6×6mm images

**DOI:** 10.1371/journal.pone.0237785

**Published:** 2020-08-21

**Authors:** Henrik Faatz, Kai Rothaus, Marie-Louise Gunnemann, Marius Book, Pia Wilming, Matthias Gutfleisch, Georg Spital, Albrecht Lommatzsch, Daniel Pauleikhoff

**Affiliations:** 1 Department of Ophthalmology, St. Franziskus Hospital, Münster, Germany; 2 Department of Ophthalmology, University of Essen–Duisburg, Duisburg, Germany; 3 Achim Wessing Institute for Imaging in Ophthalmology, University Hospital Duisburg-Essen, Duisburg, Germany; Nicolaus Copernicus University, POLAND

## Abstract

**Background:**

The aim of this study was to ascertain whether there are relevant differences between the vascular morphology of macular neovascularizations (MNV) in 3×3mm and 6×6mm images, produced by optical coherence tomography angiography (OCTA).

**Methods:**

MNV of 49 patients were automated quantitative analysed, measuring area, flow, the fractal dimension, average vessel length, vascular density, and average vessel caliber. These parameters were compared between the 3×3mm and the 6×6mm images.

**Results:**

A strong linear association was found between the 3×3mm and the 6×6mm images. While area, flow, and FD of the MNV were very similar, the 3×3mm images showed significantly lower average total vessel length, greater vascular density, and lower average vessel caliber.

**Conclusion:**

In quantitative analysis of the morphologic parameters of MNV in 3×3mm and 6×6mm images, the structures are not directly equivalent in the two sizes of scan. The images must be evaluated on an individual basis.

## Introduction

Optical coherence tomography angiography (OCTA) is a new, increasingly important imaging method that is playing an increasing role in the field of ophthalmology. Detection of the movement of red blood cells enables visualization of physiological and pathological flow in retinal and choroidal vessels. OCTA is non-invasive, in contrast to fluorescence angiography (FA), and is quicker and easier to perform. Moreover, OCTA provides an en face view of the retinal and choroidal vessels in their entirety or selectively in the segment of one’s choice [1]. On the other hand, OCTA does not deliver any information on dynamic blood flow or leakage phenomena. Since the introduction of OCTA technology, the implementation in patients with retinovascular diseases has increased every year, whereas the implementation of FA seems to have decreased in recent years, which underlines the importance of this new technology for the future [2].

FA is the gold standard for detection of macular neovascularization (MNV) in neovascular age-related macular degeneration (nAMD) [3]. Because optical coherence tomography (SD-OCT) is a non-invasive examination, it is particularly useful in cases of suspected MNV. An indication for a type 1 MNV can be shallow irregular retinal epithelium elevation (SIRE), for a type 2 MNV the subretinal hyperreflective material (SHRM) and for a type 3 MNV intraretinal edema with intraretinal hyperreflective material [4]. It is also suitable as a non-invasive monitoring of the disease course by assessing intraretinal and/or subretinal fluid, fibrosis, atrophy, and pigment epithelial detachments [5]. While the vascular morphology of MNV is difficult to assess on FA owing to the leakage phenomenon and to signal weakening due to the retinal pigment epithelium (RPE) overlying the occult portions of the lesion. In indocyanine green angiography (ICGA), however, the vascular morphology can be better evaluated, due to deeper RPE penetration because of the longer wavelengths and minimal leakage due to the molecular structure of the dye, but it can be demarcated more clearly on OCTA [6].

Furthermore, since the introduction of OCTA, MNV have also been found in intermediate AMD that show no signs of activity such as intraretinal/subretinal fluid on SD-OCT or leakage on FA, but have a 15.2-fold risk of the development of exudation [7]. This has led to the emergence of a new concept, in that we now speak, more precisely, of neovascular AMD, which is not necessarily identical with the classic notion of exudative AMD. OCTA has identified not only characteristics of vascular morphology that indicate how long the disease has existed [8], but also MNV activity criteria that correspond closely with the established criteria on FA and SD-OCT [9]. This involved precise assessment of the vascular architecture, such as the presence of a capillary vascular pattern, anastomoses, vascular arches, or the density of vessel segments.

Because the different image sizes vary in resolution, we set out to make quantitative comparisons between 3×3mm images and 6×6mm images with regard to the vascular morphology of MNV on OCTA with AngioVue (RTVue XR Avanti, Optovue, Inc., Fremont, CA, USA). Our aim was to identify any differences that might be of value in determining the comparability of vascular analyses of MNV.

## Patients and methods

The study was conducted in accordance with the Declaration of Helsinki and was approved by the ethics committee of the Westphalia–Lippe Chamber of Physicians and the University of Münster. All patients gave their written informed consent. The patients were included consecutively and the Data acquisition was retrospective.

Neovascular AMD was diagnosed for the first time in 86 eyes of 86 patients by means of FA, SD-OCT (Spectralis© HRA+OCT, Heidelberg Engineering, Heidelberg, Germany), and clinical examination. In addition, all patients underwent OCTA imaging with AngioVue (RTVue XR Avanti, Optovue, Inc., Fremont, CA, USA). Only patients with type 1, type 2, or mixed MNV were analyzed; those with type 3 MNV, polypoidal choroidal vasculopathy or aneurysmal type 1 neovascularization were excluded. Patients whose image data were of low quality (signal strength index < 50) were also excluded.

The RTVue XR Avanti obtains 70,000 scans/s with a wavelength of 840nm and penetration depth of 3mm. Each patient underwent to both 3×3mm and 6×6mm OCTA scans, consecutively, and consisting of 304x30x A-scans in order to generate two different 3D volumetric data set. The Angio Retina Custom en face slab was selected for this purpose, adjusted so that the upper margin was formed by the outer plexiform layer and the lower margin ran 60μm below Bruch’s membrane. If segmentation errors occurred, the patient concerned was excluded from analysis. In order to minimize artifacts, a projection artifacts removal (PAR) tool was applied. In the exported en-face OCTA scans we demarcated the MNV with the aid of the program Fiji (National Institute of Mental Health, Bethesda, MD, USA) and saved this MNV isolated from the rest of the image for further analysis. Intraclass correlation coefficient (ICC, 95% confidence intervals) was used to verify the consistency of the two readers (H.F. and P.W.) in the differentiation of MNV area.

The vascular network of each MNV was extracted with the aid of MatLab (Mathworks, Version R2014b). Skeletonization of the vascular network is achieved with multiscale calculation of the gradient field in the en-face image. Both extremely thin and thick vascular segments are detected as continuous midlines. The caliber (diameter) of each vascular segment is then determined, so that not only the skeletonization but also the segmentation of the network is calculated. The individual vascular segments are formed by the edges of the vascular graphs and the branching of the nodes.

**Fig 1:**
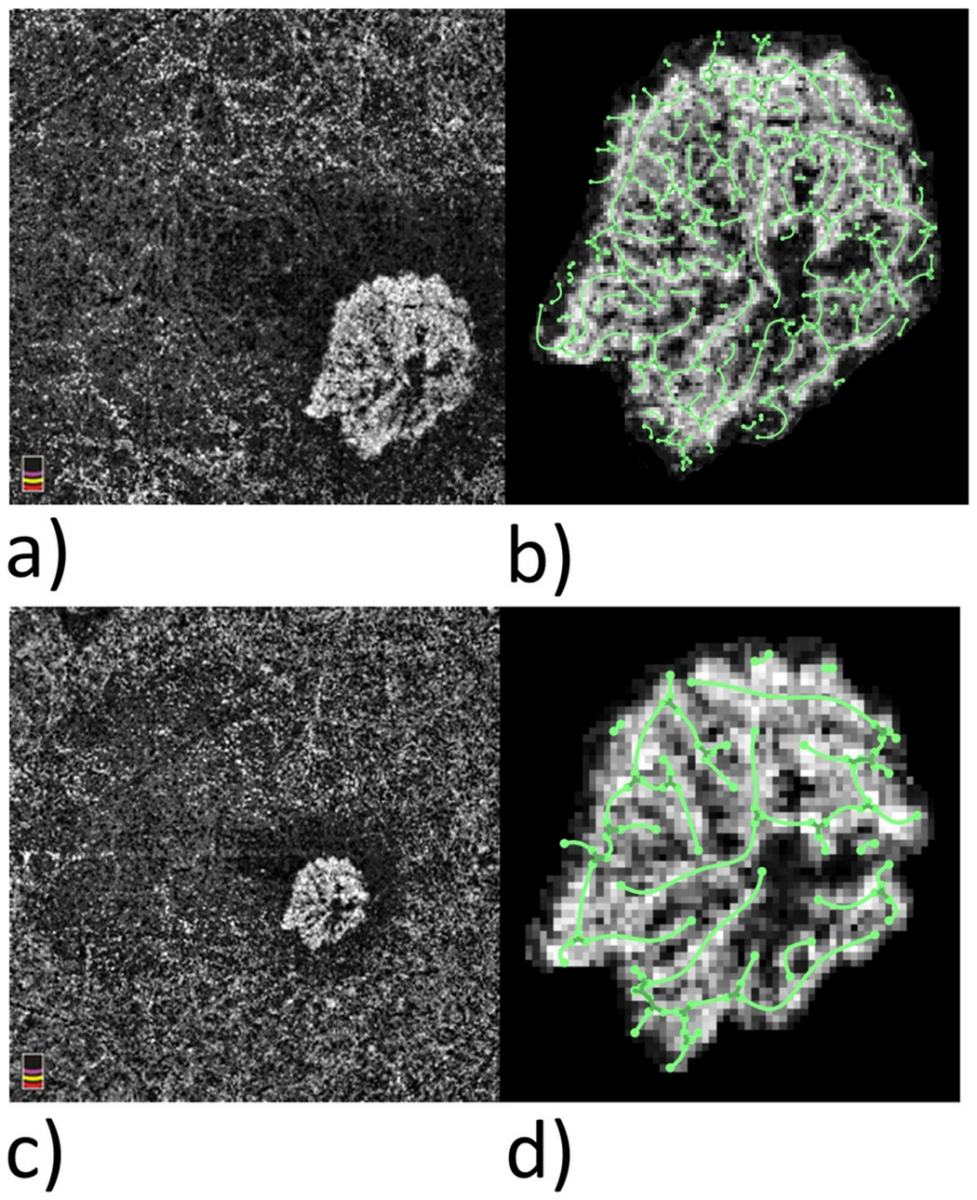
3x3mm and 6x6 OCTA images with skeletonization of the MNV. An MNV in the OCTA en face view: (a) 3×3mm image; (c) 6×6mm image; (b, d) the respective skeletonization. A 3×3mm image has a resolution of approximately 10μm/px, a 6×6mm image, 20μm/px.

In each case we compared a 3×3mm image with a 6×6mm image to find out whether the above-mentioned morphologic parameters are independent of the image resolution. Fig 1 shows an example of 3×3mm and 6×6mm OCTA images with subsequent skeletonization of the MNV.

The images are obtained in direct succession during the same examination, yielding two paired samples X and Y, which, in the ideal case, lie on the line x = y. For each morphologic parameter, we determined the correlation coefficient ρ (with the 95% data density), the p value, and the determination coefficient R^2^ of the correlation analyses. Furthermore, we calculated the gradient of the linear regression lines with the 95% data density, together with the average deviation (residual) to these lines and the average residual to the target function. To this end, we carried out a principal axis transformation. This minimizes not the summed residuals of the y-deviations (like the linear regression), but rather the euclidic distance as function of the residual [10]. Finally, we performed a Kolmogorov–Smirnov adjustment test. If a morphologic parameter is independent of image resolution, the p value of the regression analysis should lie under the 5% significance level, with the correlation coefficient and R^2^ as close as possible to 1. The gradient of the regression lines is ideally 45° and deviates only slightly from the data points for the regression lines and the target lines. The adjustment test should not be significant, as the null hypothesis X = Y is being tested.

The following parameters of the MNV were analyzed: lesion size in en face view (area), flow, fractal dimension (FD), average vascular segment length, number of vascular segments per area, and average vessel caliber.

## Results

We evaluated data from 49 eyes of 49 patients (31 women, 18 men) with untreated MNV in nAMD. Eight patients were excluded because no MNV was detected in OCTA, 4 patients had type 3 MNV and 3 had polypoidal choroidal vasculopathy and 19 patients were excluded because of poor image quality. The patients’ average age was 77.3±7.5 years and their average best corrected visual acuity was 0.52 ± 0.28 LogMAR. Assessment with FA and SD-OCT showed that 15 patients had type 1; 17, type 2; and 17 mixed type. With an ICC value of 0.964 (0.958–0.968) the agreement between the two readers regarding the delimitation of the MNV area was very strong.

The MNV area showed close correspondence (p<0.00001) between the 3×3mm and 6×6mm images, with a high correlation coefficient of ρ = 0.89, a determination coefficient of R^2^ = 0.95, and a regression line gradient of 35.09° with relative deviation of 26.96% from the target function.

The flow findings also differed non-significantly (p<0.00001) between the 3×3mm and 6×6mm scans, with a correlation coefficient of ρ = 0.89, R^2^ = 0.95, and a regression line gradient of 41.08° with a relative deviation of 15.18% from the target function.

The same was true for FD (p<0.00001), with a correlation coefficient of ρ = 0,91, R^2^ = 0.96, and a regression line gradient of 36.48° with a relative deviation of 161.49%.

In contrast, the average total vessel length of the MNV differed between the 3×3mm and 6×6mm images (p = 0.1) and there was only a weak linear association (ρ = 0.24), a determination coefficient of R^2^ = 0.79, and a regression line gradient of 9.72° with a relative deviation of 195.14%.

The number of vascular segments per area, i.e. the vascular segment density of the MNV, showed a linear association between the two image sizes (p<0.0008), but a weak correlation coefficient of ρ = 0.46, R^2^ = 0.94, and a regression line gradient of 81.80° with a relative deviation of 835.80%.

The average vessel caliber also differed between the 3×3mm and 6×6mm scans (p = 0.02), with a weak correlation coefficient of ρ = 0.34, R^2^ = 0.90, and a regression line gradient of 8.16° with a relative deviation of 622.95%.

**Fig 2:**
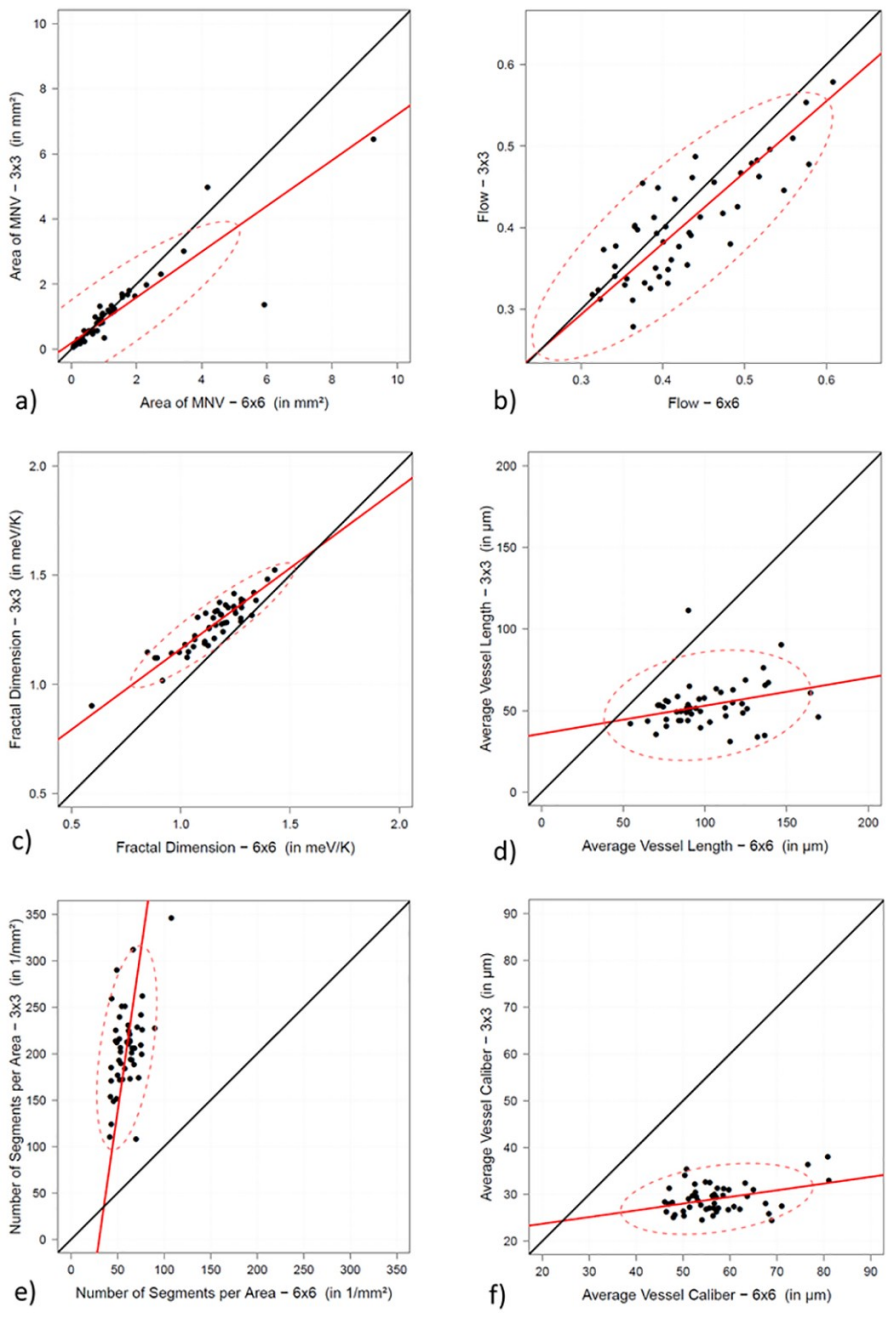
Graphical visualization of the determined vessel parameters of both image sizes. Graphs showing how the measured parameters differ between 3×3mm images (x axis) and 6×6mm images (y axis). a) Area of MNV; b) flow; c) fractal dimension; d) average vessel length; e) number of segments per area; f) average vessel caliber. Calculated linear regression line (red line); target function at x = y (black line); 95% data area (red dashed ellipse).

The results are listed in full in Table 1 with confidence intervals, residuals, and adjustment test. Fig 2 shows the acquired data points, the corresponding linear regression lines (red line), the theoretical optimal regression line at x = y (black line), and the 95% data density (red dashed ellipse) for each of the parameters measured.

**Table 1:** Overview of the analyses of the vessel parameters.

3×3mm vs. 6×6mm	p correlation	rho correlation	R^2^	RL gradient with confidence interval	Average residual RL	Average residual target function	Adjustment test: X = Y
**Area of region**	< 0.00001	0.89 [0.81, 0.94]	0.95	35.09 [31.04, 39.13]	0.0646	0.0820	1.00
**Flow**	< 0.00001	0.82 [0.70, 0.89]	0.91	41.08 [35.31, 46.84]	0.0043	0.0050	0.54
**Fractal dimension**	< 0.00001	0.91 [0.85, 0.95]	0.96	36.48 [32.87, 40.09]	0.0056	0.0145	0.0002
**Average segment length**	0.10	0.24 [-0.05, 0.48]	0.79	9.72 [-2.24, 21.67]	1.9178	5.6602	< 0.00001
**Vascular density (segments/mm**^**2**^**)**	0.0009	0.46 [0.20, 0.66]	0.94	81.80 [77.22, 86.38]	15.6731	1.6573	< 0.00001
**Average vessel caliber**	0.02	0.34 [0.07, 0.57]	0.90	8.16 [1.68, 14.64]	0.4172	3.0163	< 0.00001

Differences between 3×3mm und 6×6mm images. A linear association is indicated by a p value <0.05. The correlation coefficient with 95% confidence interval shows the strength of the linear association; R^2^ indicates how close the data points are to the calculated regression line (RL); gradient of the RL; residual to the RL; residual to the target function; the adjustment test shows whether the data are distributed in a certain way.

## Discussion

A large number of studies have described the vascular morphology of MNV [11–14]. Further subjective accounts cover especially the presence and extent of capillaries or large-caliber vessels [1, 15, 16]. Studies assessing the activity of MNV describe the vascular morphology in the active stage qualitatively as a dense, fine network with large numbers of small, branched vessels and capillaries, together with anastomoses and terminal vascular loops [9]. Significant associations between vascular morphology and disease duration have also been found: a loose vascular network is associated with long-term disease, a dense network with a recent onset [8]. We showed in a previous study that vessels can be described mathematically and that these parameters also change significantly in the course of anti-VEGF treatment [17, 18]. Detection of changes in vessels by OCTA can also make valuable contributions to the diagnosis, monitoring, and prognosis of other vascular disease, such as MacTel type 2 [19] or diabetic retinopathy [20]. OCTA’s precise three-dimensional depiction of the vessels in an MNV also plays a decisive part in interpreting the findings correctly.

A central role in comparison of published research findings is therefore occupied by the question of how quantitative OCTA analyses may vary among devices or between different scan settings (e.g. image size) on the same device. With regard to the latter, our data show close correspondence of the size of the perfused MNV area between 3×3mm and 6×6mm scans, and thus good comparability. This is in agreement with Arya et al. [21], who also found no difference in MNV size on 3×3mm and 6×6mm images using the Optovue RTVue XR Avanti and the Zeiss Cirrus HD-OCT with Angioplex. There is, however, a tendency for lesions to be estimated as larger on 6×6mm than on 3×3mm images, and the greater the actual lesion size, the higher the difference. This may be because exact demarcation around the margins is hampered by the smaller image size, and examiners tend to be generous rather than risk cutting off part of the MNV. Overall, the results, especially the adjustment test, show that the measured lesion dimensions are very closely comparable between the two sizes of scan.

The measurements of flow in the MNV also show a strong linear association between the two scan sizes, with close approximation of the regression lines to the theoretically optimal regression line. The flow values represent the proportion of all pixels that generate a detectable signal within the scanning time. The flow is dependent on a range of systemic factors (sex, age, blood pressure, exercise, drug intake, etc.) and ophthalmological variables (intraocular pressure, spherical equivalent, lens status, etc.) [22]. Since in each case the two scans compared were obtained in direct succession in the same patient, these factors probably exerted little influence. Many analyses of MNV have shown reductions in their area and flow on transition from the active to the inactive state, which indicates that this parameter is helpful in assessing the development of disease activity over time [14, 23, 24]. Nevertheless, our results indicate that 3×3mm scans show higher vessel density, smaller total vessel length, and lower vascular caliber than 6×6mm images, and that flow alone permits no conclusions as to vascular configuration; rather, the measured flow reflects various morphological features and thus may change.

The FD is a mathematical descriptor of the complexity of a structure. A higher FD is associated with a higher amount of branching in the MNV. The FD values of the two scan sizes display a high degree of linear dependence, but the FD of the 3×3mm images is always higher than that of the 6×6mm images. The significant adjustment test also shows that the two scan sizes should not be compared in this respect. The reason is that the higher resolution of the 3×3mm images depicts the vascular structure in more detail and more vascular segments are present per area. Studies have revealed a reduction in FD with anti-VEGF treatment, which speaks for a decrease in vascular branching [17, 25] and agrees with the findings reported by Spaide, who used OCTA to show that the capillary density of an MNV declines during anti-VEGF treatment, while the prominent afferent vessels show little change or none at all [1]. Since the changes in FD are very small, however, inconsistency of scan sizes during follow-up would lead to errors in the interpretation of the FD.

**Fig 3:**
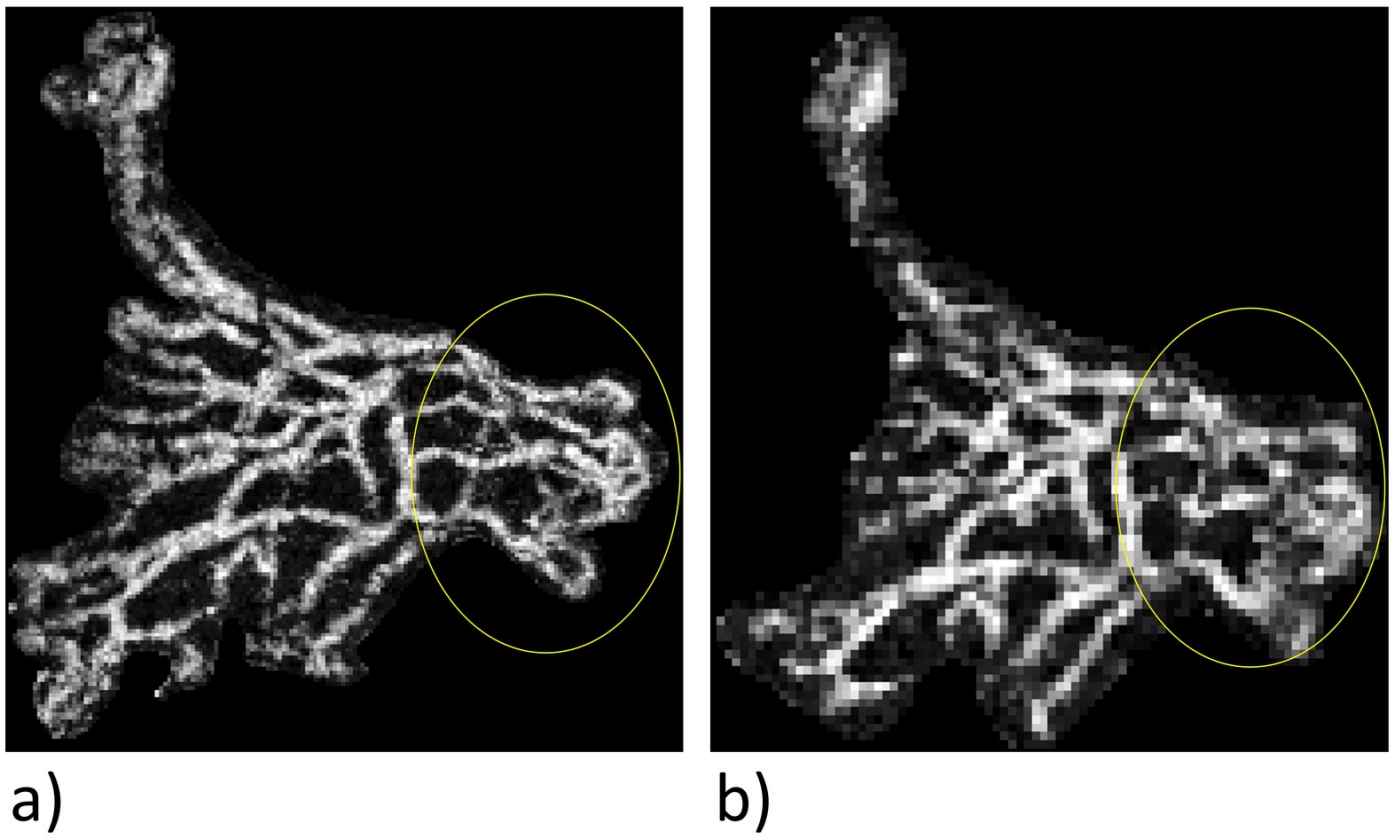
Comparison of the delimitability of fine vessels. Enlarged view of an MNV. In the area enclosed by the yellow line, there is finer, better segregated visualization of the vessels in the 3×3mm image (a) than in the 6×6mm scan (b).

The average vascular segment length is derived as follows: The algorithm records the beginning or end of a new vascular segment at every vascular intersection, branch, or ending. The average length of the segments registered is then calculated. This parameter is an indirect measure of the degree of branching in an MNV, because a spongiform vascular structure displays a shorter segment length, while a more mature vascular structure will have longer segments. We see that the average segment length is significantly greater in the 6×6mm images than in the 3×3mm scans. This may have to do with the lower resolution of the 6×6mm images, which means that fewer vascular intersections are depicted and fits in with our finding that the segment density in the 6×6mm images is much lower than in the 3×3mm images (Fig 3).

**Fig 4:**
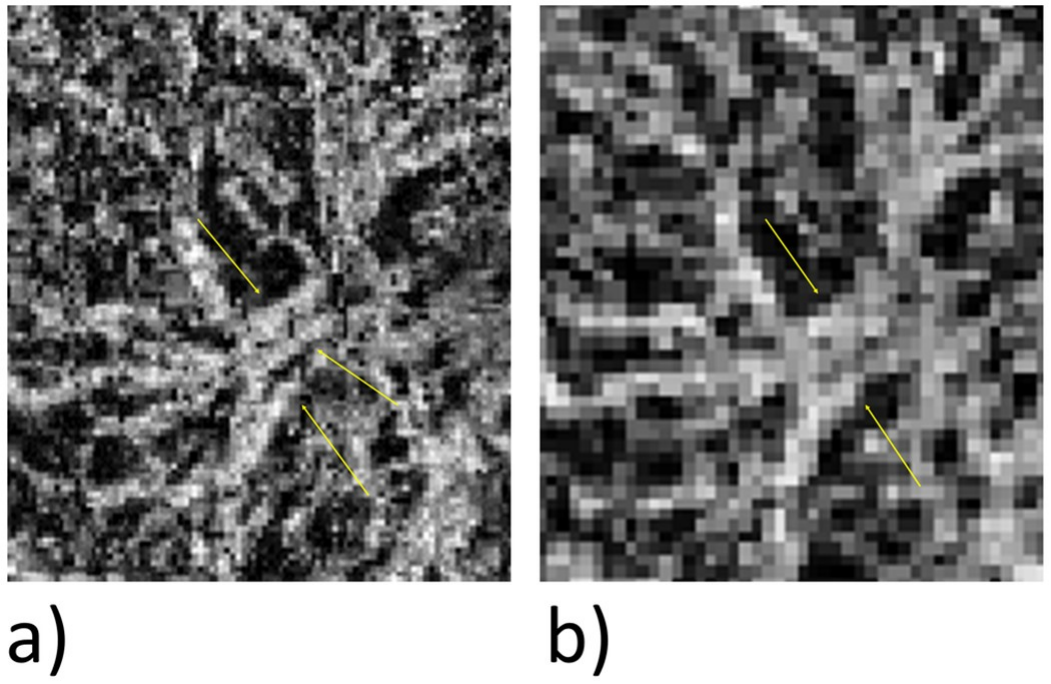
Comparison of the separability of vessels. View of an MNV. For a direct comparison of the vessel architecture, the 6x6 mm image was enlarged. As shown by the yellow arrows, three vessels are clearly differentiated from each other in the 3×3mm image (a), but two of the vessels cannot be separated in the 6×6mm scan (b).

One possible reason for this clear difference is that very fine vessels are not detected or vessels very close to one another are visualized as a single vessel, as can be seen in Fig 4.

**Fig 5:**
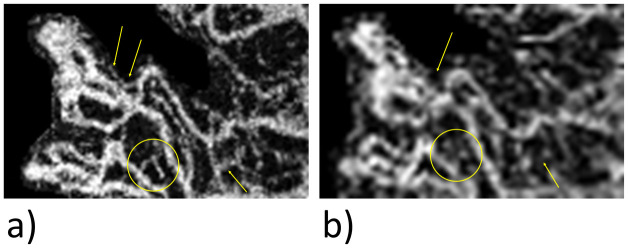
Comparison in the representation of fine vessels. Enlarged view of an MNV. The yellow circles and arrows in the 3×3mm image (a) show fine vessels that are not visualized in the 6×6mm scan (b).

The average caliber of the vessels is significantly greater in the 6×6mm than in the 3×3mm images. There are two possible reasons. First, the lower resolution means that fewer small vessels and capillaries are captured, increasing the average diameter measured (Fig 5).

Second, the inability to visualize separately vessels that lie close to each other leads to an incorrectly high assessment of vessel caliber (Fig 4). The caliber measured on OCTA scans is generally significantly higher than on fundus images, especially for low-caliber vessels [26]. Spaide et al. [27] attributed this to the fact that a signal is detected and depicted as a white pixel even if the laser beam only just touches the vessel concerned. This effect is proportionally greater in small vessels and therefore has more of an impact in lower-resolution scans.

This study features a number of limitations. First, the MNV must be manually demarcated for vascular analysis, so a certain amount of subjective variation is inevitable. Second, evaluation was restricted to MNV that could be captured in full on a 3×3mm scan. Our results therefore say nothing about the vascular structure of MNV with a larger area. Third, in future studies it will be important to include further disease entities such as type 3 NV and PCV, so that conclusions can be drawn for the entire spectrum of nAMD. Also, the diagnosis in our patient population was performed by FA and SD-OCT and not by ICGA, which allows a better assignment of MNV to its subtypes and also shows a higher sensitivity in the detection of MNV compared to OCTA [28, 29]. Fourth, the utility of the OCTA technique is restricted by shadowing artifacts caused by corneal opacity, severe cataract, retinal hemorrhage, retinal pigment epithelium, or, in principle, by any overlying retinal vascular structures. However, since the 3×3mm and 6×6mm images are obtained with no delay between them, both would be affected equally. Fifth, OCTA only captures the blood flow in a determined range of time. Variable inter-scan time analysis (VISTA) has shown that vascular morphology varies over time [30].

Although there was good agreement between 3×3mm and 6×6mm images for some parameters, such as lesion area, flow, and FD, important differences between the two scan sizes were found for average total vessel length, vascular segment density, and average vessel caliber. For purposes of clinical evaluation, this means that MNV structure as depicted on 3×3mm and 6×6mm images is not directly equivalent. Therefore, quantitative analyses of changes in an MNV over time must always be based on scans of the same size and performed with same device. Furthermore, the limited comparability of 3×3mm and 6×6mm images must always be borne in mind when comparing the published results of different studies.

The potential for quantifiable analysis of morphological changes in an MNV based on OCTA may offer new biomarkers for future licensing studies and provides additional data for possible artificial intelligence algorithms. Because of the more detailed vessel imaging in the 3x3 mm image, this is preferred for MNV imaging, but for large areas of MNV the 6x6 mm image must be used to enable complete imaging and assessment of MNV.
